# Tacrolimus-Induced Neurotrophic Differentiation of Adipose-Derived Stem Cells as Novel Therapeutic Method for Peripheral Nerve Injury

**DOI:** 10.3389/fncel.2021.799151

**Published:** 2021-12-08

**Authors:** Xiangyun Yao, Zhiwen Yan, Xiaojing Li, Yanhao Li, Yuanming Ouyang, Cunyi Fan

**Affiliations:** ^1^Department of Orthopedics, Shanghai Jiao Tong University Affiliated Sixth People’s Hospital, Shanghai, China; ^2^Shanghai Engineering Research Center for Orthopaedic Material Innovation and Tissue Regeneration, Shanghai, China; ^3^Youth Science and Technology Innovation Studio, Shanghai Jiao Tong University School of Medicine, Shanghai, China; ^4^TianXinFu (Beijing) Medical Appliance Co., Ltd., Beijing, China

**Keywords:** tacrolimus, adipose-derived stem cells, neurotrophic differentiation, nerve crush injury, peripheral nerve regeneration

## Abstract

Peripheral nerve injuries (PNIs) are frequent traumatic injuries across the globe. Severe PNIs result in irreversible loss of axons and myelin sheaths and disability of motor and sensory function. Schwann cells can secrete neurotrophic factors and myelinate the injured axons to repair PNIs. However, Schwann cells are hard to harvest and expand *in vitro*, which limit their clinical use. Adipose-derived stem cells (ADSCs) are easily accessible and have the potential to acquire neurotrophic phenotype under the induction of an established protocol. It has been noticed that Tacrolimus/FK506 promotes peripheral nerve regeneration, despite the mechanism of its pro-neurogenic capacity remains undefined. Herein, we investigated the neurotrophic capacity of ADSCs under the stimulation of tacrolimus. ADSCs were cultured in the induction medium for 18 days to differentiate along the glial lineage and were subjected to FK506 stimulation for the last 3 days. We discovered that FK506 greatly enhanced the neurotrophic phenotype of ADSCs which potentiated the nerve regeneration in a crush injury model. This work explored the novel application of FK506 synergized with ADSCs and thus shed promising light on the treatment of severe PNIs.

## Introduction

Peripheral nerve injury (PNI) is a frequent clinical issue which affects the overall quality of life. Nerve crush injuries in peripheral nerves lead to long-term functional disability in patients. The regeneration process requires restoration of microenvironment, including angiogenesis, immune homeostasis and oxidative stress rectification (Höke, 2006; Scheib and Höke, 2013; Qian et al., 2018a, 2019d). The intrinsic regenerative capacity of peripheral nerves fails in severe injuries, leading to axonal and myelin sheath disruptions (Yan et al., 2021). Therefore, strategies for optimized nerve repair after severe crush injuries are extensively sought for.

Over the past years, Schwann cell (SC) transplantation has emerged as possible strategy to assist in the clinical treatment after PNIs (Guénard et al., 1992). On the one hand, SCs produce neurotrophic factors, such as brain derived neurotrophic factor (BDNF), glial-cell derived neurotrophic factor (GDNF), nerve growth factor (NGF) and neurotrophin-3 (NT-3), which facilitate the axonal regeneration and neurite outgrowth (Makwana and Raivich, 2005). On the other hand, SCs wrap axons and are crucial for the formation of myelin sheath (Salzer, 2015). However, the requirement for autologous nerve donor and the poor proliferation ability limits the application of SCs in the clinical practice (Lehmann et al., 2012). In this background, advanced pharmacological therapies have centered on the neurotrophic action of drugs to improve clinical outcomes of nerve repair (Gold et al., 1999; Moosavi et al., 2016).

Adipose derived stem cells (ADSCs) have potential to alternate SCs in translational medicine (Lehmann and Höke, 2010, 2016). ADSCs can be easily harvested from autologous fat tissue and expanded *in vitro*. Their plasticity of differentiating into SC-like cells (SCLCs) was demonstrated both *in vitro* and *in vivo* (Dezawa et al., 2001; Kingham et al., 2007; Xu et al., 2008). According to the established protocol, a differentiation cocktail consisting glial growth factors could induce rat ADSC into SCLCs. The most common differentiation cocktail to induce ASCs differentiation includes 5 ng/mL platelet-derived growth factor (PDGF), 10 ng/mL basic fibroblast growth factor (bFGF), 14 μm forskolin and 200 ng/mL glial growth factor-2 (GGF-2). Such treatment to some extent recaptures the feature in the nerve microenvironment, as axons could secret large amount of GGF-2 to stimulate the neurotrophic function of SCs (Birchmeier and Nave, 2008). After 18 days of induction culture *in vitro*, SCLCs not only exhibited SC phenotypes, but also gained the functional properties of SCs such as myelinating ability and stimulation of neurite outgrowth (Kingham et al., 2007; Xu et al., 2008). Nevertheless, the efficiency to induce the differentiation of ADSCs remains unsatisfactory due to the relatively low rates of differentiation and the short duration of neurotrophic phenotype maintenance (Sun et al., 2018).

The immunosuppressive drug tacrolimus/FK506 has been demonstrated to promote peripheral nerve regeneration (PNR) in the clinical application due to their excellent anti-inflammatory performance (Cooney and Hentz, 2001; Jablecki et al., 2009). In recent years, the neurotrophic actions of FK506 have received more and more attention and the underlying FK506-binding protein (FKBP), which is abundantly expressed in neurons, has been proved to activate the downstream pathway responsible for the neurotrophic actions (Daneri-Becerra et al., 2020). In the present study, we explored the neurotrophic potential of FK506 synergized with ADSCs in a rat nerve crush injury model. We demonstrated that the neurotrophic phenotype was enhanced in ADSCs due to the addition of FK506. We propose that the transplantation of FK506-preconditioned ADSCs can compensate the lack of SCs in the treatment of severe PNIs.

## Materials and Methods

### Cell Culture

Rat ADSCs were obtained from Cyagen Biosciences (RASMD-01001, Guangzhou, China). ADSCs were incubated with monolayer cultures, including α-MEM (Invitrogen, Thermo Fisher Scientific, Waltham, MA, United States), 10% fetal bovine serum (FBS) (No. 11885-084; Gibco) and 1% (v/v) penicillin–streptomycin solution (Sigma-Aldrich, Poole, United Kingdom). ADSCs were maintained in T75 flasks (Corning Ltd., United Kingdom) at 37°C, 5% CO2 and 95% humidity. Culture media were changed every 3 days and cell monolayer was split when subconfluent.

Neuron-like pheochromocytoma (PC12) cells were purchased from the Cell Bank of Chinese Academy of Sciences (Shanghai, China). The cells were cultured in specific culture medium to prevent spontaneous differentiation. The PC12-specific culture medium contains 5% horse serum (Beijing Solarbio Science and Technology Co., Ltd.), 5% FBS, 100 U/mL penicillin and 100 μg/mL streptomycin.

### Cell Viability

The cell viability following the treatment of FK506 was determined by CCK-8 assay. Briefly, we seeded undifferentiated ADSCs in 96-well plates at a concentration of 5000 cells per well. To investigate how different concentrations of tacrolimus affected ADSCs, stem cells were incubated with 1 control (no tacrolimus) and 4 different tacrolimus concentrations (1, 10, and 100 ng/mL, 1 μg/mL). The concentration of FK506 was established with the administration of cell culture medium, which was also used to dilute tacrolimus. Following 24, 48, or 72 h of culture, the supernatant was carefully aspirated and the cells were rinsed with Phosphate Buffered Saline (PBS) (D8537, Sigma-Aldrich, Shanghai, China) prior to incubation in a 10% (v/v) solution of CCK-8 reagent (Dojindo, Japan) diluted in α-MEM (10 μL CCK-8 reagent was added to each 100 μL of medium) and cells were incubated at 37°C for 1 h. The absorbance at 450 nm was measured with a micro-plate reader. All the experiments were repeated at least three times.

### Glial Lineage Differentiation

At passage 2, ADSCs which were previously plated in 6 well plates at 60% confluency were chemically induced to a Schwann-like phenotype using an established protocol consisting in three steps (Supplementary Figure 1): (1) 24 h treatment with 1 mM β-mercaptoethanol (Sigma-Aldrich, Poole, United Kingdom); (2) wash with PBS solution (D8537, Sigma-Aldrich, Shanghai, China) followed by 72 h treatment with 35 ng/mL all-trans-retinoic acid (A506728, Sangon Biotech, China); (3) exposure for 14 days to a differentiation cocktail consisting in α-MEM supplemented with 5 ng/mL PDGF-AA (Peprotech EC Ltd., London, United Kingdom), 10 ng/mL bFGF (Peprotech EC Ltd., London, United Kingdom), 14 mM forskolin (Sigma- Aldrich, Poole, United Kingdom), and 200 ng/mL GGF-2 (Acorda Therapeutics Inc., Ardsley, NY, United States). After 4 and 10 days of exposure to the differentiation cocktail, cells were split and replated in six- well plates to allow expansion and differentiation.

Platelet-derived growth factor-AA is a subtype of PDGF and promotes the proliferation of Schwann cells by activating Erk pathways (Ogata et al., 2004). Forskolin, as a cAMP-elevating agent, enhances the gene expression of NGF (Abukhashim et al., 2011). The treatment of bFGF could up-regulate the transcription of brain-derived neurotrophic factors within axons, which promote axonal regeneration (Soto et al., 2006). GGF-2 is axon-derived neuregulin which has trophic effects on Schwann cells in terms of proliferation, migration, myelination and neuron-glia interactions (Mahanthappa et al., 1996; Kopp et al., 1997). The combined application of these growth factors orchestrates the differentiation and proliferation of Schwann cells (Jessen and Mirsky, 1999).

### Characterization of Differentiated Adipose-Derived Stem Cells by Immunofluorescence

Adipose-derived stem cells were seeded on coverslips covered with poly-L-lysine (No. P8920; Sigma) in 6-well plates and the immunofluorescence method was applied to show SC associated biomarkers.

After incubation for 24 h, the medium in the wells was removed. Coverslips with attached cells were then washed with 1 × PBS. The cells were then fixed with 4% paraformaldehyde for 10 min. After washing three times with PBS for 5 min each time, ADSCs were blocked with 10% goat serum (SL038; Solarbio) in PBS for 1 h. Primary antibody anti-S100β (1:200, ab52642, Abcam, United States); It was dropped onto coverslips and incubated at room temperature overnight. Next, secondary antibody, Goat Anti-Rabbit IgG H&L (Alexa Fluor^®^ 594; 1:200; ab150084; Abcam), was dropped onto coverslips. After incubation with secondary antibodies in the dark for 1.5 h, the coverslips were washed and incubated with DAPI (No. H-1200; Vector Laboratories Inc., Burlingame, CA, United States). For the final step, the coverslips were inverted onto the slides and analyzed under a fluorescent microscope. The number of surviving cells, and the rates of S100β - positive cells in the 5 groups of ADSCs were calculated according to 5 randomly selected fields in each group at 200 × magnification using IPP 6.0.

### RNA Extraction and Quantitative Real−time PCR

After incubation with FK506 for 3 days, the supernatant was carefully aspirated and the cells were rinsed with PBS for three times. Then, the total cellular RNA was extracted with an EZ-press RNA Purification Kit (B0004DP, EZBioscience^®^), and reversely transcribed into cDNA with Color Reverse Transcription Kit (with gDNA Remover) (A0010CGQ, EZBioscience^®^) according to the manufacturer’s instructions. Real-time PCR amplification was performed using the Step One Plus Real-Time PCR System (Applied Biosystems, Thermo Fisher Scientific, Waltham, MA, United States) by the following procedure: first at 95°C for 5 min, and then 40 cycles of 95°C for 15 s and 60°C for 60 s. The primer sequences of the neurotropic factors and glial markers are listed below. The relative quantification of gene expression was analyzed with the values of 2^–ΔΔCt^, and normalized by GAPDH expression level. All detections were repeated three times separately.

**Table:** 

Gene	Forward (5′–3′)	Reverse (5′–3′)
NGF	TCATCCACCCACCCAGTCTTCC	TCCGTGGCTGTGGTCTTATCTCC
BDNF	TGGTCAGTGGCTGGCTCTCATAC	TCAGAACAGAACAGAACAGAACAGGAC
GDNF	CGCTGACCAGTGACTCCAATATGC	AGTGCCGCCGCTTGTTTATCTG
NT3	GACAGTCCAAGTTATATCGCAGTGAGG	ACTCCGTCCGAATGAAGGTGTTATTG
MBP	AGAGAAACGCAGGGACGAAACTTC	GCAAGGTCGGTCGTTCAGTCAC
GAPDH	ATGGTGAAGGTCGGTGTGAACG	TTACTCCTTGGAGGCCATGTAG

### Western Blot Assay

After 18 days of glial induction culture and 3 days of FK506 stimulation, ADSCs were lysed in RIPA lysis buffer to collect their total proteins. Next, sodium dodecylsulfate polyacrylamide gel electrophoresis (SDS-PAGE) was performed, and the protein samples were transferred onto PVDF membranes. These PVDF membranes were then incubated at 4°C overnight with the anti-myelin basic protein (MBP) (1:1000, ab40390, Abcam, United States) and GAPDH (1: 1500, ab8245, Abcam), following by incubation at room temperature for 2h with goat anti-rabbit IgG-HRP (1: 1000, 33101ES60). Signals were developed using ECL™ Western Blotting Analysis System (Sigma-Aldrich, United States) and Image J v1.46 software was used to develop signals.

### Enzyme-Linked Immunosorbent Assay

Differentiated ADSCs (1.25*10^5^) were seeded into 96-well plates and maintained in normal culture medium for 24 h without the addition of FK506 and glial growth factors. The supernatant was then collected and analyzed by ELISA using the Rat NGF ELISA kit (NO. SDR0016, Simuwubio^®^) and Rat GDNF ELISA kit (NO. SDR0037, Simuwubio^®^), according to the manufacturer’s protocol. All samples were analyzed in triplicate, and the absorbance was measured at 450 nm on a microplate reader (Tecan, Inc.). The quantity of NGF and GDNF (pg/mL) were calculated against standard curves that were produced using recombinant proteins provided in the kits and normalized to the final cell counts after 24 h of incubation.

### Pheochromocytoma Cells Co-culture With rADSCs in a Transwell^®^ System

Pheochromocytoma cells were seeded in the lower chambers (1 × 10^4^ cells/well) of a Transwell^®^ system (3450; Corning) with six inserts, 24 mm in diameter with 0.4-μm pore size polyester membrane in six-well plates. The FK506- pretreated differentiated ADSCs were resuspended and seeded in the upper inserts of the Transwell^®^ system at a density of 1 × 10^6^ cells/insert and incubated at 37°C, 95% humidity and 5% CO2. DMEM supplemented with 5% horse serum, 10% FBS, 100 U/mL penicillin and 100 μg/mL streptomycin was used to co-culture the different ADSCs and PC12 cells. Molecules secreted by the ADSCs were able to permeate to the PC12 cells without direct cell contact.

After co-culture for 3 days, the neurotrophic effect of ADSCs on the PC12 cells was evaluated. PC12 cells co-cultured with undifferentiated ADSCs were set as control. PC12 cells with axons longer than the average cell diameter were used for data analysis. After 3 days of co-culture with ADSCs, two separate parameters were used to quantify the ability of ADSCs in the promotion of neurite outgrowth: percentage of cells extending neuritis and length of longest neurite.

### Adipose-Derived Stem Cell Transplantation

Nine Sprague-Dawley (SD) male rats (weighing 300–350 g) were equally allocated into 3 groups for *in vivo* experiments, in accordance to the “3R rule” (reduction, replacement and refinement) for animal welfare postulated by Russel and Burch in 1959 (Russell and Burch, 1959). All the animal experiments were conducted in accordance with guidelines approved by Institutional Animal Care and Use Committee of Shanghai Jiao Tong University affiliated Shanghai sixth people’s hospital (DWSY2020-0232). Initially, 3 rats of each group were housed in the same cage. All the rats were placed under general anesthesia using intra-peritoneal injections of 3% sodium pentobarbital. The surgical site was shaved and cleaned. For the duration of the experimental protocol, anesthesia was maintained, and all of the animals were subjected to a right sciatic nerve crush procedure using sterilized microsurgery instrument. We used Corning^®^ Matrigel^®^ High Concentration (HC) (Cat No. 354248, Supplementary Figure 2) as cell loading platform in the present study. The details of the sciatic nerve crush procedure were as follows:

The right thigh was fixed and the overlying skin was incised and retracted. Then the superficial fascia was bluntly dissected. To expose the sciatic nerve, a 1–2-cm longitudinal incision was made through the gluteal muscle using microsurgery instruments. The nerve was crushed with fine forceps for 30 s as previously described (Bridge et al., 1994). 1 μL of Matrigel was injected through the epineurium into the injury site with a Hamilton microsyringe. The rats were randomized into three groups: a control group (*n* = 3) subjected to Matrigel injections without cells, a FK506-treated group (*n* = 3) subjected to Matrigel injections with FK506-treated differentiated ADSCs and a non-treated group (*n* = 3) subjected to Matrigel injections with non-treated differentiated ADSCs. In the ADSC transplantation groups, around 10^6^ of differentiated ADSCs was suspended in 1 μL Matrigel and administered into the epineurium using the Hamilton microsyringe. In the control group 1 μL of Matrigel was applied by perineural injection. In the FK506-treated groups, a concentration of 1 μg/mL FK506 dissolved in α-MEM was applied to cells for 3 days before injection, but no FK506 was added in the ADSC-suspended Matrigel. The nerves were harvested and processed for histology.

### Walking Track Analysis and Sciatic Function Index

The motor function recovery after crush injury was evaluated using a walking track and footprint analysis. At 3 weeks after cell transplantation, the hind paws of the rats were stained with a dark dye and allowed to walk in a walking track covered with white paper. The rat footprints were thus collected and analyzed. The sciatic function index (SFI) was calculated based on the previous equation (de Medinaceli et al., 1982):

Sciatic Function Index = –38.3 (PLExp – PLCtrl)/(PLCtrl) + 109.5 (TSExp – TSCtrl)/(TSCtrl) + 13.3 (ITSExp – ITSCtrl)/(ITSCtrl).

PL represents the print length (longitudinal length) and TS represents the distance from the first to fifth toe. ITS indicates the distance from the second to the fourth toe. The length was measured for an experimental (exp) or control (ctrl) foot. Three animals per group and three footprints per rat were measured, from the normal and experimental feet. Data analysis was performed following a blinding procedure as described in supporting information.

### Electrophysiology

Electrophysiological assessment was carried out after walking track analysis. Two variables, the nerve conduction velocity (NCV) and distal compound muscle action potential (DCMAP) were assessed at week 3 post-transplantation. Before starting to record, right thigh was fixed and the overlying skin was incised and retracted. The sciatic nerve was carefully exposed with proper anesthesia. In this study, we conducted our electrophysiological analysis via a bipolar stimulation electrode. As reported previously (Rodríguez Sánchez et al., 2019; Zhu et al., 2020), one electrode was positioned at the proximal and distal end of the sciatic nerve for electricity transduction. Another electrode, was inserted in the belly of the gastrocnemius muscle to record the NCV and DCMAP. We measured the distance between the electrodes and recorded and compared the peak value of DCMAP and NCV with a digital electromyography device on the gastrocnemius muscle among different groups.

### Histological Evaluation of Regenerated Sciatic Nerve Following Cell Transplantation

All the rats were sacrificed by intraperitoneal injection of a sodium pentobarbital overdose at 3 weeks following cell transplantation. The sciatic nerve was clearly exposed and excised. Consistent with previous literatures (Qian et al., 2018b, 2021b), we carried out measurements on the same portion of each nerve and same distance from the point of injury (5 mm distal to the site of crush lesion site). Cross-sections of each sciatic nerve midpoint were cut into 5-μm thick transverse sections on a cryostat. These histological sections were then placed in Sodium Citrate Antigen Retrieval Solution (C1032; Solarbio) for 10 min at 95°C. The sections were then transferred to PBS containing 10% goat serum for 30 min at room temperature after washing three times with PBS for 5 min each time. The primary antibodies anti-MBP (1:100, ab40390, Abcam, United States) and anti-β III tubulin antibody (1:100, ab7751, Abcam, United States) were used to incubate sections in a humidified chamber overnight at 4°C. Next morning, Goat Anti-Mouse and Goat Anti-Rabbit IgG H&L secondary antibodies were applied to the sections and incubated in the dark for 1 h at room temperature after rinsing off excess primary antibodies with PBS. After washing three times with PBS, the nuclei were counterstained with DAPI. Five randomly selected fields of each group were captured to analyze the regenerated axons and remyelinated nerve fibers by Image J software.

The regenerative nerves located at the distal ends of the nerve grafts were also fixed to cut transverse sections at a thickness of 7 μm using a glass knife in an ultramicrotome (EM UC7; Leica). The paraffin-embedded sections were stained with Hematoxylin-Eosin (H&E) staining solution (ab245880, Abcam). The stained sections were observed under 400× magnification, and 5 randomly selected fields were captured for each sample to calculate the area and mean density of myelinated nerve fibers by Image J.

### Histological Evaluation of the Gastrocnemius

The gastrocnemius muscle of injured site was harvested and fixed in 4% formaldehyde for 2 h. Transverse paraffin sections of the gastrocnemius muscle belly 10 μm thick were cut and stained with HE staining solution (ab245880, Abcam). Five randomly selected fields in each group at 200 × magnification were captured to measure the density and mean cross-sectional area of the gastrocnemius fibers by IPP 6.0.

### Statistical Analysis

All data are calculated and presented as the means ± standard deviation (SD) from three and more independent experiments in each group. Statistical analysis was performed using SPSS (version 22.0; IBM). We have performed student’s *t* test to compare differences between two groups, and one-way analysis of variance (ANOVA) to compare differences between multiple groups. Differences between groups were considered significant at ^***^p < 0.001, ^**^p < 0.01, and ^*^p < 0.05.

## Results

### *In vitro* Culture of Adipose-Derived Stem Cells and Schwann Cell-Like Cells

**FIGURE 1:**
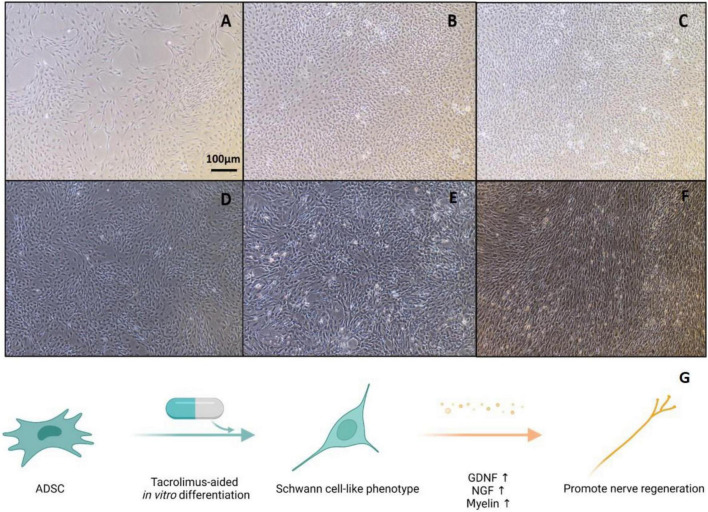
Representative graphs of undifferentiated ADSCs and Schwann-cell like ADSCs. **(A)** Cultures of undifferentiated ADSCs showed a fibroblast-like morphology. **(B)** Differentiated ADSCs adopted a spindle elongated SC-like shape. **(C–F)** In the treatment of 1, 10, 100, and 1000 ng/mL FK506, ADSCs did not exhibit evident morphological changes compared with non-treated ADSCs. Scale bar = 100 μm. **(G)** Schematic illustration of experimental design. ADSCs were first cultured in SC culture medium for 18 days and subjected to FK506 stimulation in the last 3 days of this differentiation procedure.

In this study, we cultured ADSCs in glial differentiation-induction medium following the previously established protocol (Kingham et al., 2007). Then, we added FK506 into the conditioned medium in the last 3 days of the induction culture period. Undifferentiated ADSCs are adherent cells characterized with fibroblast-like shape (Figure 1A). After 4 days of pre-conditioning and 14 days of stimulation with a mixture of growth factors (forskolin, bFGF, PDGF-AA, and GGF-2), a large number of ADSCs became morphologically similar to SCs, exhibiting a spindle-like bipolar or tripolar shape (Figure 1B). The differentiated ADSCs with fibroblast-like morphology were still occasionally observed and a small fraction of these cells retained rounded cell bodies with multiple processes. The number of surviving cells in differentiated ADSCs was slightly decreased, after the addition of FK506 in the last 3 days of induction culture. The addition of FK506 did not change the number or morphology of the differentiated ADSCs (Figures 1B–F). A schematic representation can best describe this procedure (Figure 1G).

**FIGURE 2:**
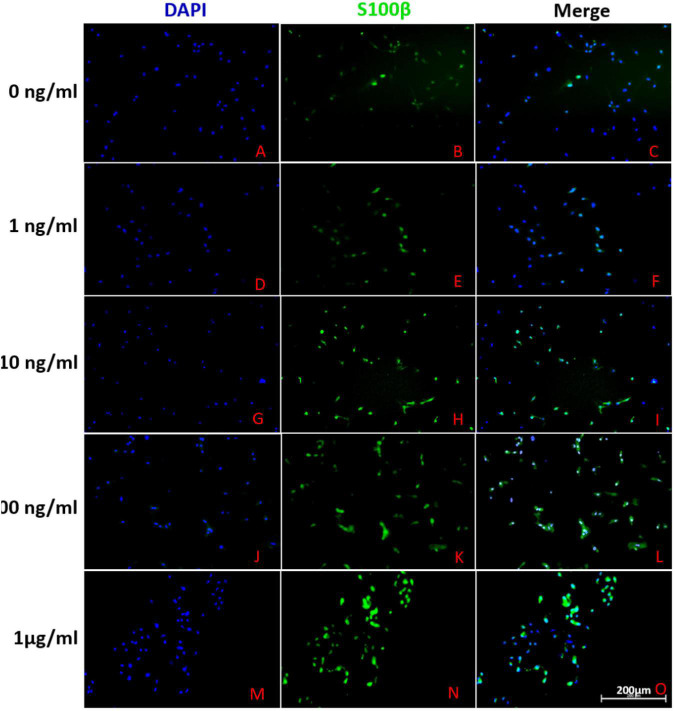
Representative immunofluorescence photographs of differentiated ADSCs derived from different groups. **(A–C)**, **(D–F)**, **(G–I)**, **(J–L)** and **(M–O)** described the S100 expression in ADSCs treated with 0, 1, 10, 100 and 1000 ng/ml FK506 respectively. The leftmost column of figures represented cell nuclei stained with blue fluorescence of DAPI. In the second column, the green fluorescence corresponded to S100-positive cells. The rightmost column of figures represented the merge of the two fluorescence from same area. Scale bar = 200 μm.

Adipose-derived stem cells were stained with S100β, a special biomarker of SCs, to evaluate its glial differentiation (Figure 2). Under the induction of differentiation cocktail, a portion of ADSCs (40.01 ± 4.14%) were positive for S100β. However, the rate of S100-positive cells did not significantly increase or decrease under the addition of 1, 10, 100, and 1000 ng/mL FK506 (42.64 ± 2.27, 46.53 ± 2.32, 50.54 ± 2.14, and 47.35 ± 3.54% respectively). These results indicated that the addition of FK506 did not change the glial differentiation of ADSCs significantly.

**FIGURE 3:**
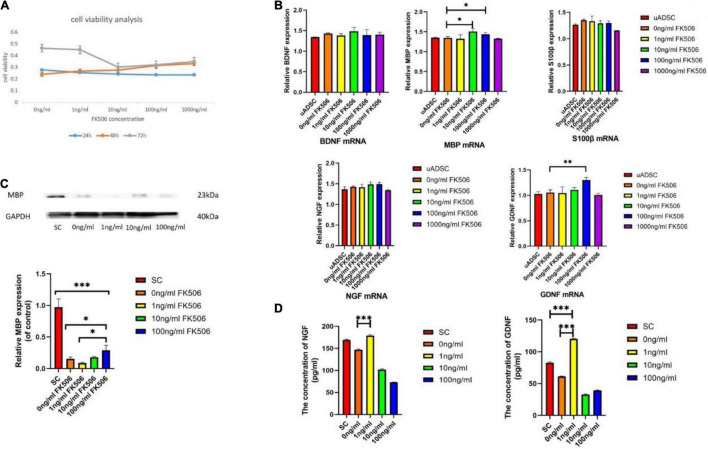
**(A)** Effects of different doses of FK506 on ADSC viability. Data are presented as the mean value ± SD from triplicate experiments. **(B)** Effects of FK506 on the mRNA expression of S100β and neurotrophic factors in differentiated ADSCs. Undifferentiated ADSCs were set up as negative control. The genes expression was assessed by quantitative real-time PCR after treatment of FK506 for 3 days. Data are presented as the mean value ± SD from triplicate experiments. **(C)** Western blot results for MBP expression. SC lysates were set up as the positive control group. Three independent replicates were included for each group. GAPDH was used as a loading control. The relative MBP expression of control was analyzed; *p < 0.05, ^**^p < 0.01, ^***^p < 0.001, compared to the 100 ng/mL FK506 treatment group; **(D)** The levels of NGF and GDNF secreted by ADSCs. SC supernatants were set up as the positive control group. ^***^P < 0.001, compared to the 0 ng/mL FK506 treatment group.

To assess the cytotoxicity of FK506, we compared the cell viability of ADSCs treated with different doses of FK506 varying from 1, 10, and 100 ng/mL and 1 μg/mL. As shown in Figure 3A, there was no significant difference among the 5 groups in the first 24 and 48 h. However, after 72h, cell viability of ADSCs treated with 10, 100, and 1000 ng/mL FK506 significantly decreased compared to that of the control group (0 ng/mL) (*p* < 0.05 at 10, 100, and 1000 ng/mL).

### mRNA and Protein Expression

We compared the glial differentiation potential of ADSCs in terms of gene expression level. After 18 days of induction culture, the relative mRNA expression levels of S100β, MBP, BDNF, GDNF, and NGF within ADSCs were examined by qRT-PCR (Figure 3B). Consistent with the outcome of immunocytochemistry, the relative expression level of S100β mRNA was not significantly different among different groups, indicating the negligible effect of FK506 on glial differentiation. MBP is the major component of myelin sheath which wraps around axons and is critical to the electrical conduction of peripheral nerves (Liu et al., 2019). The gene expression level of MBP was increased in response to the stimulation of 10 and 100 ng/mL FK506. This phenomenon was further confirmed in Western blot analysis (Figure 3C) that the MBP expression was higher in ADSCs treated with 10 and 100 ng/mL FK506 compared with other groups.

Apart from the initiation of myelination, neurotrophin secretion is another important function of SCs. It is well established that GDNF and NGF are the most critical factors for the regrowth of motor neurons and sensory neurons respectively (Johnson et al., 2015). BDNF has similar effect on neurons and provides synergistic pro-neurogenic effect when combined with other neurotrophic factors. In this regard, the relative mRNA expressions of neurotrophins, including NGF, BDNF and GDNF, were evaluated. The mRNA expression of GDNF was significantly enhanced in response to the stimulation of 100 ng/mL FK506. There was no significant difference in NGF and BDNF mRNA expression level between different groups. It should be emphasized that as the concentration of FK506 increased to 1 μg/mL, the secretion of neurotrophins has decreased on the contrary. Therefore, the highest concentration of FK506 might inhibit the cell viability and function of ADSCs. An optimal concentration of FK506 would promote the greatest extent of cellular activities, which is important for further evaluation of cellular functions. Therefore, the concentration of 1 μg/mL FK506 was not selected for the following evaluation (Western blot and ELISA analysis).

### Secretion of Neurotrophins

It has been proved in the knock-out mice that these two neurotrophins synergize in repairing the injured nerves through common intracellular signaling pathways (Airaksinen and Saarma, 2002). Therefore, after the gene expression analysis, we investigated the NGF and GDNF concentrations in ADSC culture supernatants. The results showed that ADSCs produced significantly higher levels of GDNF (120.28 ± 0.84 pg/mL) after treatment with 1 ng/mL FK506, whereas treatment with other concentrations of FK506 caused smaller increases in GDNF production (Figure 3D). The neurotrophin secretion of SCs was measured as a positive control. A similar tendency was also found for NGF. In the dADSCs treated with 1 ng/mL FK506, NGF secretion levels (178.63 ± 2.10 pg/mL) showed significantly greater enhancement than that in the other groups.

### Differentiated Adipose-Derived Stem Cells Exerted Trophic Effect on PC12 Cells

**FIGURE 4:**
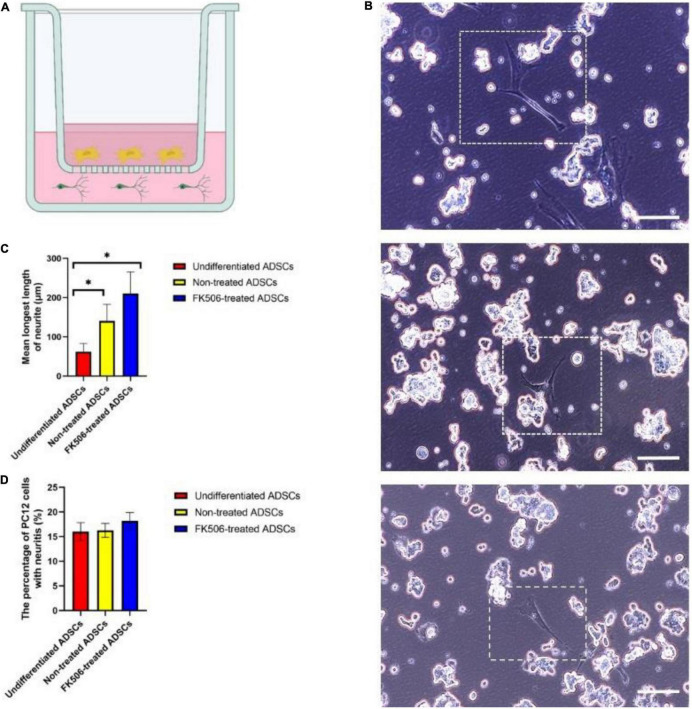
**(A)** Schematic diagram of the ADSC and PC12 cells indirect co-culture system. ADSCs were seeded in the upper chamber while PC12 cells were seeded in the lower chamber. ADSCs could not migrate across the porous polyester membrane but the neurotrophic factors secreted by ADSCs could pass freely through the inserts. **(B)** Representative images of PC12 cells co-cultured with ADSCs after 72 h. From the top to the bottom, PC12 cells were co-cultured with FK506-treated ADSCs, non-treated ADSCs and undifferentiated ADSCs. Scale bar = 100 μm. **(C,D)** The percentage of PC12 cells with neuritis and the average length of longest neurite were calculated on PC12 cells co-cultured with undifferentiated ADSCs, non-treated ADSCs and FK506-treated ADSCs. Data are resented as mean ± SD. *P < 0.05 Data from differentiated ADSCs was significantly different compared with undifferentiated ADSCs.

Schwann cells promote neurite outgrowth through neuronal-glial bidirectional signaling. The establishment of reciprocal interaction between ADSCs and neuronal cells is the premise for ADSCs to develop neurotrophic phenotype. In this regard, we examined the ability of ADSCs to stimulate neurite outgrowth in the model of PC12 cell, a neuron-like cell line which retains the characteristics of dopaminergic neurons (Genchi et al., 2015). We used a Transwell co-culture system to study the effects of ADSCs on PC12 cells (Figure 4A). Neurotrophins trigger neurite projections *in vitro* and peripheral nerve innervation *in vivo* (Avwenagha et al., 2003; Schmidt and Rathjen, 2010). According to the ELISA analysis result, we selected the 1 ng/mL FK506-treated ADSCs as the experimental group. Undifferentiated PC12 cells are non-adherent and tend to gather in clusters. Therefore, neurite extension can only be analyzed in adherent PC12 cells. PC12 cell differentiation was determined 72h after the treatment of FK506. The neurites longer than soma diameters were analyzed in PC12 cells (Figure 4B).

After 3 days of co-culture, only a small fraction of PC12 cells have extended neurites (Figure 4B). Although differentiated ADSCs exhibited significantly more neurotrophic effect than their undifferentiated counterpart in terms of the length of longest neurite on PC12 cells (*p* < 0.05) (Figure 4C), there was no significant difference in response to FK506. The addition of FK506 did not evoke an increase in the percentage of PC12 cells with neuritis (*p* < 0.01) (Figure 4D).

### Functional Recovery and Electrophysiology of the Sciatic Nerve Following Cell Transplantation

To compare the neurotrophic effect of ADSCs *in vivo*, we performed nerve crush injuries and measured the different regenerative response of peripheral nerves after ADSC transplantation.

**FIGURE 5:**
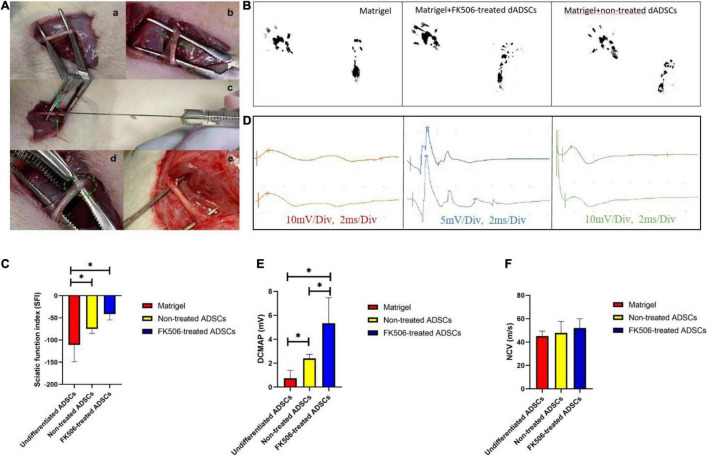
**(A)** Sciatic nerve injury model and Matrigel administration. The crushed site and injection site were indicated by green arrow and green circle respectively. (a) Sciatic nerve exposure. (b) Crush injury. (c) Matrigel injection through microliter syringe. (d) Matrigel in the epineurium. (e) Sciatic nerve was exposed again at 3 weeks post-injection. **(B)** Representative footprints of rats treated with Matrigel, FK506 treated ADSCs and non-treated ADSCs. **(C)** Gait analysis after cell transplantation. The improvement of the SFI in FK506-treated ADSC group was significantly superior (*p* < 0.05) to those in the non-treated groups. Both cell transplantation group showed significant improvement compared with the Matrigel control group. Data are expressed as means ± SD. *P < 0.05, compared with non-treated ADSC transplantation group. **(D)** Representative oscillograms of each group at 3 weeks post-surgery. **(E,F)** The DCMAP and NCV evaluation in different groups. *P < 0.05 DCMAP data from FK506-treated and non-treated dADSC group was significantly higher compared with Matrigel group.

Nerve crush injuries cause damage to all axons and myelin sheaths but the epineurium and basal lamina remain intact (Bridge et al., 1994). In this context, we transplanted ADSCs into the epineurium of crushed site through direct injection (Figure 5A). Matrigel is known to be compatible with the peripheral nerve microenvironment and can completely degrade over time, which allows for the gradual release of loaded cells (Huang et al., 2019). Therefore, we administered ADSC suspension into rats in the form of cell-embedded Matrigel. After nearly 3 weeks of cell transplantation, functional recovery of the sciatic nerve was evaluated by gait analysis.

Our *in vitro* studies have identified that 1 ng/mL FK506 exerted better neurotrophic potential on ADSCs in comparison with other concentrations. Therefore, we only studied the efficacy of 1 ng/mL FK506-treated differentiated ADSCs *in vivo*. The rats were placed on a walking track 3 weeks after Matrigel injection to collect footprints for SFI scoring (Figure 5B). SFI scores were calculated in each rat as previously described (Lopez-Silva et al., 2021). The mean SFI scores were −74.62 ± 10.54, −41.66 ± 12.86, and −110.77 ± 38.11 in the non-treated ADSCs, FK506-treated ADSCs and Matrigel control groups respectively (Figure 5C). The ADSC transplantation groups had significantly better reparative effect in comparison with Matrigel injection group. Besides, statistically significant differences between the ADSC transplantation groups were observed at 3 weeks post-injury (*p* < 0.05).

Electrophysiological evaluation of each group was performed at 3 weeks post-operation, to evaluate the functional restoration of sciatic nerves. Representative electrophysiological oscillograms of each group were shown in Figure 5D. The DCMAP and NCV were induced and recorded. The results of electrophysiological analysis revealed a slight increase of DCMAP and NCV in response to the transplantation of ADSCs (Figures 5E,F). The DCMAPs of both ADSC transplantation group (5.3 ± 2.1 and 2.4 ± 0.3 mV respectively) were notably higher than that of the control group (1.1 ± 0.5 mV, *p* < 0.05), but no significant difference was seen between the two ADSC transplantation groups.

### Histological Evaluation of Regenerative Nerves Following Cell Transplantation

Nerve crush injury is a common model in the peripheral nerve studies. Despite there are various crushing methods, the severity of injury was similar in terms of histology, in which Wallerian degeneration and axonotmesis were observed (Bridge et al., 1994).

**FIGURE 6:**
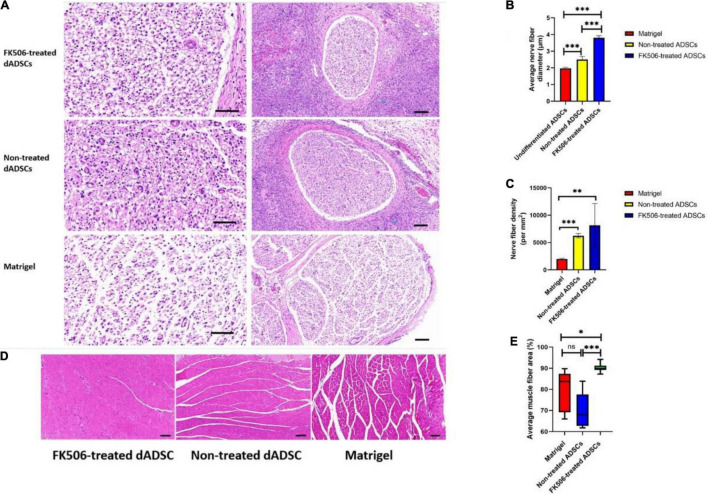
**(A)** Histological evaluation of regenerated nerves at 3 weeks after cell transplantation. The scale bar in left column figures is 50 and the right is 100 μm. **(B,C)** The nerve fiber diameter and density evaluation in different groups. ^**^P < 0.01, ^***^p < 0.001. **(D)** Representative HE staining results of the gastrocnemius showed muscle fibers and the collagen fibers distributed among them. Scale bar 100 μm. **(E)** The muscle fiber area in different groups. *P < 0.05, ^***^p < 0.001.

In our study, all samples were dissected from 15 mm sections of regenerated nerves distal to the injury site at 3 weeks postoperatively. HE staining was performed to validate the morphological improvement in the different groups. The results for the Matrigel group (A), FK506-treated ADSC transplantation group (B), and non-treated ADSC transplantation group (C) were displayed (Figure 6A). After 3 weeks of recovery, the relatively tenuous nerve fibers together with a lot of fibrous tissue were found in the Matrigel groups. Counting the number or density of nerve fibers is a commonly used method to evaluate the status of regenerated nerves between different groups (Qian et al., 2020a). However, during the peripheral nerve regeneration, nerve fibers tend to branch at the site of the lesion, leading to the increase of fiber numbers in the regenerated distal nerves (Wood et al., 2011). Therefore, the increase of fiber number alone is not sufficient to prove the maturation of regenerated nerves. The diameter of nerve fibers was then calculated to back up the measurement of nerve maturation (Cheng et al., 2019). Moreover, the diameter of nerve fibers is also related to the reconstruction of electroactive properties, due to the fact that NCV increases along with the increase of fiber diameter (Hasegawa and Komiyama, 1993). In this context, we evaluated the density and average diameter of myelinated fibers in the HE-stained sections of regenerated sciatic nerves under a light microscope (Leica, United States) (Figure 6A).

In general, the regeneration outcome of crushed nerves in FK506-treated ADSC transplantation group was remarkably better than that in the other two groups. The mean diameter of myelinated nerve fibers in the FK506-treated ADSC group was significantly larger than that in the non-treated ADSC group and Matrigel group (Figure 6B). The fiber density in FK506-treated ADSCs group and the non-treated ADSCs group was much higher than that in the Matrigel group (Figure 6C). The density of nerve fibers showed similar trends as the averaged fiber diameter which indicated the optimal regeneration outcome of crushed nerve in FK506-treated ADSC transplantation group.

**FIGURE 7:**
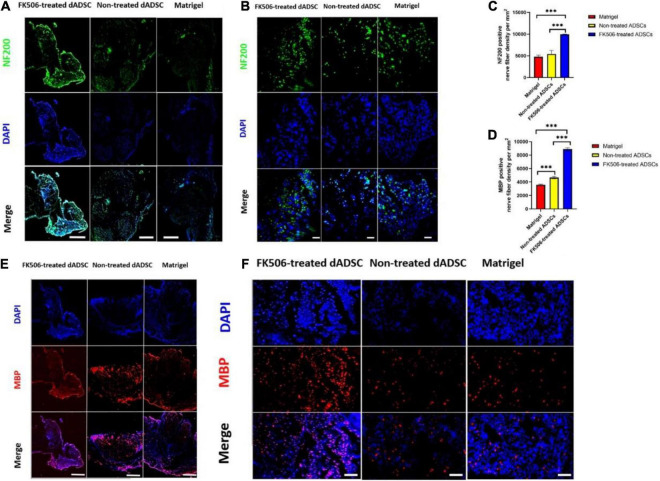
**(A,B)** The immunofluorescence (IF) photographs of the regenerated axons. NF-200 positive axons were stained with green fluorescence. The nuclei were stained with blue fluorescence. Scale bar is 50 μm for **(A)** and 10 μm for **(B)**. **(C,D)** The mean density of regenerated axons which were NF-200 positive and the mean density of myelinated nerve fibers which were MBP-positive. ^***^P < 0.001. **(E,F)** The IF photographs of the myelinated nerve fibers. MBP positive axons were stained with red fluorescence. The nuclei were stained with blue fluorescence. Scale bar is 50 μm for **(E)** and 20 μm for **(F)**.

Although HE is the most commonly used stain, it is not an adequate method for nerve tissue staining because the myelin sheaths are not labeled and they are thus hard to be detected. Therefore, green fluorescence labeled NF200 – positive axons (Figures 7A,B) and red fluorescence labeled MBP – positive myelin sheaths (Figures 7E,F) were observed as an additional histological evaluation. In our results (Figures 7C,D), the mean density of regenerated axons labeled with green fluorescence and the mean density of myelinated nerve fibers labeled with red fluorescence were significantly higher (*p* < 0.001) in FK506-treated ADSC transplantation groups, than those in the non-treated ADSC group and Matrigel group.

Representative HE staining outcomes of the gastrocnemius are also shown here to evaluate the structural recovery of denervated muscles (Figure 6D). Both ADSC transplantation groups showed the hyperplasia of collagen fibers which were distributed among muscle fibers. However, the degree of gastrocnemius atrophy was alleviated. The mean cross-sectional area of gastrocnemius fibers in the FK506-treated ADSC group was significantly larger than those in the non-treated ADSC group and Matrigel group (Figure 6E).

## Discussion

Peripheral nerve regeneration relies on the recovery of neural structure and remodeling of imbalanced neuronal microenvironment (Jiang et al., 2020; Qian et al., 2021a,c; Yao et al., 2021a). The inhibition of excessive immune responses is of vital importance to restoring the balance in microenvironment after PNIs (Qian et al., 2019b, 2020a,b). The treatment of long-gap nerve defects requires highly functional biomimetic scaffolds to bridge the proximal and distal stumps (Qian et al., 2019a,c; Chen et al., 2020; Yan et al., 2020; Yang et al., 2020). In contrast, the treatment of severe nerve crush injuries relies more on the pharmacotherapy. Therefore, the application of immunosuppressant drugs has arisen in the treatment of various neuropathies, due to their rapid control of inflammation. Major advances in pharmacological therapies have noticed the phenomenon that immunosuppressive drug tacrolimus promotes peripheral nerve regeneration, but the cellular and molecular biological mechanism of its pro-regenerative capacity remain undefined. On the one hand, tacrolimus alleviates the injury-induced inflammation in the peripheral nerves, as a classic immunosuppressive agent. On the other hand, tacrolimus directly accelerates axonal regeneration via the inhibition of calcineurin activity, leading to the increased phosphorylation of growth associated protein 43 (GAP43) (Gold et al., 1995). More importantly, FK506 and its derivatives manipulate the gene expression of neurotrophins in SCs. SCs critically affect the regeneration of axons and myelin through the secretion of neurotrophin and myelin sheath following crush injury (Gaudet et al., 2011). These neurotrophins induce multiple intracellular pathways that boost the regeneration of peripheral nerves. The direct neuroregenerative effect of FK506 was revealed by utilizing the non-immunosuppressive derivative of FK506 which potentiated the glial-neuronal signaling during regeneration (Birge et al., 2004).

Adipose-derived stem cells have considerable promise in the clinical treatment of PNIs (Yao et al., 2021b). Autologous fat tissue is easily accessible via liposuction or abdominal incision. Fat tissue contains a higher density of mesenchymal stem cells than bone marrow tissue and ADSCs are shown to proliferate more rapidly than those from other tissues (Strem et al., 2005). Besides, ADSCs have multi-lineage differentiation potential and differentiate along the glial lineage when cultured in conditioned medium (Strem et al., 2005; Orbay et al., 2012; Qian et al., 2018). The most commonly used protocol for the induction of ADSCs in glial differentiation is the Kingham method (Kingham et al., 2007). However, this method exhibited unsatisfactory efficiency due to the lengthy induction duration and relatively low differentiation rate. ADSCs rapidly dedifferentiated in the absence of glial differentiation culture medium (Faroni et al., 2016). Therefore, when ADSCs are applied to compensate the lack of SCs after severe PNIs, the neurotrophic functional phenotype is more valuable than the SC-like structural phenotype. Considering the above, we brought up the novel use of tacrolimus to stimulate neurotrophic phenotype of ADSCs. In the present study, we explored the neurotrophic differentiation and peripheral nerve repair potential of ADSCs under the stimulation of FK506. To examine the efficacy of FK506 in different doses, we divided the ADSCs into 5 groups: non-treated ADSCs and ADSCs treated with 1, 10, 100, and 1000 ng/mL FK506.

Although apoptosis of ADSCs was observed in our study during the 18 days of differentiation process, both FK506-treated and non-treated ADSCs cultured in induction medium showed excellent capacity of differentiation toward SCs. Spindle- shaped dADSCs was observed which was close to the morphology of mature SCs. At 18 days after glial induction, immunofluorescence (IF) staining showed that some of ADSCs had differentiated into S100-positive cells. However, there was no significant difference in the rate of S100-positive cells between ADSCs treated with different doses of FK506. The results of qRT-PCR were consistent with this outcome that FK506 exerted no significant impact on the structural differentiation of ADSCs evaluated by glial markers. During the development of mature Schwann cells, pluripotent stem cells (PSCs), neural crest stem cells (NCSCs), Schwann cell precursors (S) and immature Schwann cells appear in a successive manner (Jessen and Mirsky, 2005). In this background, NCSC-specific markers (e.g., PAX3, TWIST, and SLUG), NCSC- and SCP-specific markers (e.g., FOXD3, NGFR, and SOX10), and SCP-specific markers (e.g., CDH19, GAP43, and MPZ) are sequentially expressed along the differentiation (Kim et al., 2017). However, the differentiation of ADSCs enjoys totally different pattern. As a matter of fact, the SC- like trans-differentiation of ADSCs is transient which does not include regular developing process (Faroni et al., 2016). Therefore, the Schwann cell-like differentiation of ADSCs is simply the phenotypic transformation. Based on these facts, we did not examine the expression of neural intermediate markers. Furthermore, Schwann cells of different developmental stage could be characterized by different markers. Krox-20, O1, MAG, P_0_, CNPase, Prx, and PMP-22 are frequently used as markers of the differentiated, myelinating Schwann cell phenotype, while GFAP, c-Jun, p75^NGFR^, N-Cadherin, nestin, and vimentin are used as markers of the immature, non-myelinating (Soto and Monje, 2017). Noteworthy, S100β and O4 (sulfatide) can be used as general Schwann cell-specific markers. In the present study, we tested the expression of S100β to measure the Schwann cell-like differentiation of ADSCs. It is very true that we can hardly say or judge the differentiation efficiency of ADSCs simply via S100β. As the previous study mentioned, many Schwann cell markers (e.g., GFAP, P75NGFR, MPZ, and S100β) can be employed to characterize Schwann cell phenotype, but all of these markers had low expression (less than 15%) following the established neural induction protocol (Ramli et al., 2019). The accurate characterization of Schwann cell-like differentiation may require more rigorous assessments (including but not limited to Schwann cell specific markers and transcription factors listed above).

Schwann cells are known to produce a lot of neurotrophins, such as NGF, BDNF, NT-3, and GDNF. These neurotrophins target at different subsets of dorsal root ganglion (DRG) neurons and act cooperatively on the neurite outgrowth and axonal regeneration. FK506 interacts with FKBP and alters the gene expression in SCs, which might be responsible for the enhanced release of neurotrophins. ADSCs also have the capacity to secrete various neurotrophins which contribute to its pro-neurogenic effect (Suganuma et al., 2013). In this background, we hypothesized that the neurotrophic potential of differentiated ADSCs can be enhanced by FK506. Our qRT-PCR results showed that the level of BDNF, GDNF, NGF mRNA expression increased in ADSCs under the stimulation of FK506. Furthermore, the stimulation effect was magnified when FK506 was administered in proper concentrations. The neurotrophin stimulation effect of FK506 was further demonstrated by ELISA analysis. GDNF and NGF are two major neurotrophins that are significantly more effective than other growth factors in promoting axonal regeneration, and a synergistic effect of NGF and GDNF was observed in the treatment of peripheral nerve injury (Xiao et al., 2012). We found that FK506 induced considerable increase in GDNF and NGF secretions. Consistent with the results of qRT-PCR, this stimulation effect was optimized in a specific concentration of FK506, indicating the concentration-dependent neurotrophic effect of FK506. Secretion of neurotrophic factors by SCs can attract neurite outgrowth and guide axons to target reinnervation. A PC12 cell co-culture system was established to determine the effect of FK506-treated ADSCs on neurite elongation *in vitro*. PC12 cells are commonly used neuron-like model for the analysis of axon and neurite regeneration (Zhou et al., 2013). In our Transwell system, PC12 cells cocultured with the FK506-treated ADSCs exhibited longer axon length than the other two groups in the Transwell^®^ system. This result implied that FK506-treated ADSCs had greater potential to stimulate axonal regeneration.

Schwann cells form myelin sheaths around axons which promise the rapid conduction of bioelectricity. It has been previously revealed in histomorphometric assessment that administration of low-dose FK506 had positive effect on the myelination of injured sciatic nerves. The myelin sheath secretion of ADSCs was also measured in transcriptional and translational level. Accordingly, the treatment of 10 and 100 ng/mL FK506 created better stimulation efficacy as enhanced mRNA and protein expression level of MBP were observed.

In general, our *in vitro* study has confirmed that the neurotrophic phenotype of ADSCs was enhanced by FK506 in terms of neurotrophin release and myelin sheath secretion while the morphology and structure were not affected as measured by glial markers. The myelin-forming ability of Schwann-cell like ADSCs has been studied with similar *in vitro* protocols in a previous study (Xu et al., 2008). The glial differentiation was identified by S100β, GFAP and P0 immunofluorescence, and the myelin-forming capacity was confirmed via PC12 co-culture system. However, this study mainly focused on the myelination capacity, without paying attention to other neurotrophic function of differentiated ADSCs, including neurotrophic factor secretion. The *in vivo* myelination capacity was also unexplored. A previous study also combined the application of mesenchymal stem cells and local tacrolimus delivery to promote nerve regeneration (Saffari et al., 2021). However, this research centered on the anti-inflammatory effect rather than the neurotrophic action of tacrolimus in cell and nerve graft transplantation.

Compared with transection of nerves, crush injury also leads to complete disruption of the axons and their myelin sheath, but the basal lamina sheaths around individual axon/SC units are intact. The remaining basal lamina tube allows SCs to align in columns (bands of Bungner) which enclose the regenerating axons and enable axons to reconnect with their target tissues. To extend the *in vitro* studies and assess whether FK506 might also affect axonal regeneration, remyelination and nerve function recovery *in vivo*, we also investigated the FK506-treated ADSC transplantation in a sciatic nerve crush model.

Matrigel is a class of natural material that mimics the structure and components of extracellular matrix (ECM), which creates a permissive environment for nerve regeneration and reinnervation from the proximal side of the injury (Xu et al., 2020). Corning^®^ Matrigel^®^ is the solubilized basement membrane extraction prepared from Engelbreth-Holm-Swarm (EHS) mouse sarcoma, a tumor rich in ECM proteins. Different from other widely used cell loading system (e.g., hydrogel and fibrin), Matrigel is composed of laminin, collagen IV, heparan sulfate proteo-glycan and entactin. Therefore, the product is not only non-immunogenic but also effective for cellular attachment and differentiation. According to the manufacturer’s instruction, when given in high concentration (18–22 mg/mL), Matrigel keeps the injected cells localized for *in situ* analysis. In this context, ADSCs could be delivered locally by Matrigel to the crushed nerve site. We prepared the FK506-treated and non-treated ADSCs in ice-cold Matrigel to make cell suspension and injected them directly into the injury site. After a comprehensive review on the protocols of several previous researches, around 10^6^ of differentiated ADSCs was suspended in 1 μL Matrigel and then administered into the epineurium of the injured site through direct injection (Hasegawa and Komiyama, 1993; Chen et al., 2007).

In the existing literature, the fate of the transplanted ADSCs remains unelucidated. The survival of transplanted cells depends on multiple composite factors in the nerve microenvironment, including new vessel formation, energy metabolism and inflammatory regulation (Chen et al., 2013; Gao et al., 2018; Mokhtari-Jafari et al., 2020). Therefore it could be extremely hard to tell the exact number of cells left in the transplanted site. Burdick et al. suggested the viability of transplanted neural stem cells in a stroke model could be lower than 10% (Burdick et al., 2016). So far, there was no quantitative study that reported the survival of transplanted cells in sciatic nerve injury model. In a previous study, Zhu et al. constructed the olfactory ensheathing cell (OEC)-loaded nerve conduit, in which green fluorescent protein (GFP)-expressing OECs were suspended in fibrin gel and seeded uniformly in the conduit (Zhu et al., 2014). They analyzed the cell viability within nerve conduit at 3, 7, 14, and 28 days after transplantation. The number of GFP-expressing OECs within nerve conduits has decreased significantly at each time point following transplantation. On the twenty-eighth day after surgery, the number of GFP-expressing OECs has been reduced by half compared with that on the third day. Despite the great loss of viable ADSCs after being transplanted *in vivo* for a long time (Erba et al., 2010), ADSCs could exert their neuro-regenerative function at the early stage via secretion of neurotrophic factors or paracrine crosstalk to Schwann cells (Georgiou et al., 2015; Ching et al., 2018; Bucan et al., 2019; Rodríguez Sánchez et al., 2019; Jiang et al., 2021). Jiang et al. claimed that the transplanted cells could improve regeneration outcomes as long as more than 10^6^ cells survive (Jiang et al., 2021). A previous study reported the direct myelination of differentiated ADSCs in a peroneal nerve-denervated rat model (Tomita et al., 2012). Accordingly, the researchers labeled Schwann cell-like ADSCs with CM-Dil and transplanted these cells *in vivo*. At 10 weeks after cell injection, immunofluorescence analysis revealed the CM-Dil fluorescence co-localized with MBP and P0 protein in the regenerated peroneal nerve, which indicated the expression of myelin protein in ADSCs. Moreover, both GFAP positive cells (29.1%) and GFAP negative, S100β positive cells (8.1%) were frequently observed among the CM-Dil positive cells in the peroneal nerves, indicating the presence of both non-myelinating and myelinating ADSCs. Similarly, Tomita et al. also proved the direct myelination of ADSCs in the rat sciatic nerve model (Tomita et al., 2013). In another study, GFP-labeled neural crest stem cells (NCSCs) were pre-induced into Schwann cell precursors before loading onto the nerve scaffolds. At 5 weeks after *in vivo* transplantation, most GFP-expressing cells in the nerve defect expressed SC markers S100β and P0, which demonstrated that NCSCs differentiated into SCs *in vivo* (Li et al., 2021a). Similarly, Li et al. transplanted GFP-labeled NCSCs to transected spinal cord via seeding NSCs onto collagen scaffolds (Li X. et al., 2016). Two months post injury, many GFP-expressing NSCs were also positive for NeuN, Tuj1 and Map2 neuronal markers, which indicated that NSCs differentiated into neurons *in vivo*. Collectively, these data confirmed that the transplanted ADSCs were dictated by the host microenvironment and their neurotrophic phenotype could be maintained in the nerve microenvironment. However, the outstanding neurotrophic effect of differentiated ADSCs might be limited to the early stage of nerve regeneration due to the low cell survival rate.

The *in vivo* reconstruction of motor function was evaluated by electrophysiological test and footprint analysis from walking behavior test. At week 3 following cell transplantation, the SFIs in the FK506-treated and non-treated ADSC transplantation group were significantly lower than those in the Matrigel groups. Although FK506-treated ADSCs secret more neurotrophins *in vitro*, there was no significant improvement compared with non-treated ADSC group in the analysis of walking behavior. This might be attributable to the higher level of neurotrophin secretion in FK506-treated ADSCs, which stimulated the proximal axonal stump of sensory neuron in sciatic nerves. Such stimulation resulted in severe pain in the limb and thus reduced the touching duration of this limb on the ground (Apfel, 2000). Therefore, in order to understand the recovery of motor function in sciatic nerve, we tested the neural electrophysiological evaluation *in vivo* as a complement. At week 3 after cell transplantation, the amplitude of DCMAP in the FK506-treatment group was superior to those of the other groups, suggesting the better improvement in nerve bioelectricity conduction. The myelination and axonal regeneration were assessed by immunofluorescence (IF) and histological techniques. Consistent with the results of *in vitro* experiments, IF analysis of the regenerated nerves further confirmed that the density of the regenerated axons and remyelinated nerve fibers were higher in FK506-treated ADSC transplantation group than those in the other groups. Sciatic nerve innervation is critical to the maintenance of skeletal muscle structure and function. The denervation of axons can lead to rapid muscle-fiber atrophy within the first 2 weeks after nerve injury (Engel and Stonnington, 1974; Yue et al., 2019). Therefore, we performed the quantitative study on the morphology of gastrocnemius to see the recovery of muscle denervation. The cross-sectional areas of gastrocnemius fibers in rats transplanted with FK506-treated ADSCs were superior to those in the other groups.

In addition to their neurotrophic potential, differentiated ADSCs have a number of non-neurotrophic effects that also promote nerve regeneration, including anti-fibrotic effects. In fact, accumulating studies have noticed the great anti-fibrotic effect of ADSCs on hypertrophic scar fibrosis which is mediated through the paracrine function of ADSCs. Yang et al. discovered that conditioned medium (CM) of ADSCs induced the apoptosis and inhibited the proliferation of keloid dermis-derived fibroblasts (KFBs) via the activation of the COX-2/PGE2 cascade in KFBs (Yang et al., 2021). Li et al. reported the decrease of collage deposition and scar formation in skin fibroblasts under the treatment of ADSC-CM or ADSC-exosomes (Li Y. et al., 2016; Li et al., 2021b). Similarly, Chai also revealed that treatment of ADSC-CM decreased the expression of collagen I, collagen III, and α-smooth muscle actin within myofibroblasts via p38-MAPK pathway (Chai et al., 2019). Therefore, ADSCs can exert anti-fibrotic effect via paracrine function, although the detailed functioning molecules remains unelucidated. FK506 is recognized as a useful immunosuppressive and immunomodulatory drug in the treatment of auto-immune disease and transplantation rejection in the clinical avenue (Broen and van Laar, 2020). However, there is no existing literature that revealed its function in anti-fibrosis. On the contrary, fibrotic nephrotoxicity and pulmonary fibrosis are both complications of tacrolimus treatment (Goldfarb, 2003). Therefore, FK506 may have no direct anti-fibrotic effect in the process of peripheral nerve regeneration. Therefore, inhibition of fibrosis in the nerve regeneration procedure is not a contributing factor for the FK506-promoted nerve regeneration.

Improved surgical techniques, low-frequency electrical stimulation and local FK506 administration are the most frequently used interventions to optimize the clinical outcome of nerve repair (Gordon, 2020). Although the therapeutic effect of FK506 as traditional adjuvant therapy has been clinically proven, the adverse effects when systemically administered have restricted its broader application in PNIs (Zuo et al., 2020). Local delivery to the injured site with various biocompatible devices could prevent the complication of FK506 systematic administration and appears to have great translational promise (Davis et al., 2018; Tajdaran et al., 2019). Preclinical assessment of local FK506 delivery devices has centered on the advancement of their clinical translatability, in terms of biocompatibility, effectiveness and producibility (Zuo et al., 2020). Herein, we brought up a new protocol to exert the neurotrophic effect of FK506 on ADSCs, and thus avoided the side effect of FK506 administration. Considering the fact that autologous ADSCs are easy to harvest and have been applied in clinical avenue for years (Banyard et al., 2015), our protocol possess great translational promise as a novel therapeutic method.

## Conclusion

In conclusion, we established a SC-like ADSC model to explore the therapeutic effect of FK506. ADSCs were induced into SCLCs and stimulated with FK506. These cells displayed better neurotrophic properties and myelinating capacity. *In vivo*, FK506-treated ADSC transplantation significantly enhanced axonal regeneration and functional recovery following sciatic nerve injuries. Our research demonstrated that SC-like ADSCs stimulated by FK506 acquired better performance in the peripheral nerve regeneration.

## Data Availability Statement

The original contributions presented in the study are included in the article/Supplementary Material, further inquiries can be directed to the corresponding author/s.

## Ethics Statement

All the animal experiments were conducted in accordance with guidelines approved by the Institutional Animal Care and Use Committee of Shanghai Jiaotong University Affiliated Shanghai Sixth People’s Hospital (DWSY2020-0232).

## Author Contributions

CF and YO conceptualized the study. XY, ZY, XL, and YL participated in the study design, searched databases, extracted and assessed studies, and helped draft the manuscript. XY, ZY, YO, and CF wrote and revised the manuscript. All authors read and approved the final version.

## Conflict of Interest

XL was employed by company TianXinFu (Beijing) Medical Appliance Co., Ltd. The remaining authors declare that the research was conducted in the absence of any commercial or financial relationships that could be construed as a potential conflict of interest.

## Publisher’s Note

All claims expressed in this article are solely those of the authors and do not necessarily represent those of their affiliated organizations, or those of the publisher, the editors and the reviewers. Any product that may be evaluated in this article, or claim that may be made by its manufacturer, is not guaranteed or endorsed by the publisher.

## Abbreviations

ADSC: adipose-derived stem cell
BDNF: brain derived neurotrophic factor
bFGF: basic fibroblast growth factor
CM: conditioned medium
DCMAP: distal compound muscle action potential
DRG: dorsal root ganglion
ECM: extracellular matrix
FKBP: FK506-binding protein
FBS: fetal bovine serum
GDNF: glial-cell derived neurotrophic factor
GGF-2: glial growth factor-2
GFP: green fluorescent protein
GAP43: growth associated protein 43
HE: hematoxylin-eosin
IF: immunofluorescence
MSC: mesenchymal stem cell
MBP: myelin basic protein
NGF: nerve growth factor
NT-3: neurotrophin-3
NCSCs: neural crest stem cells
NCV: nerve conduction velocity
OEC: olfactory ensheathing cell
PNIs: peripheral nerve injuries
PNR: peripheral nerve regeneration
PC12 cells: pheochromocytoma cells
PBS: phosphate buffered saline
PDGF: platelet-derived growth factor
SFI: sciatic function index
SC: Schwann cell
SCLCs: Schwann cell-like cells
SD: Sprague-Dawley.
