# Supplementary material for: Tacrolimus-Induced Neurotrophic Differentiation of Adipose-Derived Stem Cells as Novel Therapeutic Method for Peripheral Nerve Injury
Source: Front Cell Neurosci. 2021 Dec 8;15:799151. doi: 10.3389/fncel.2021.799151 (PMC8692949; doi:10.3389/fncel.2021.799151)
Supplement: Supplementary file 1 [file Data_Sheet_1.docx]

**Supplementary Information**


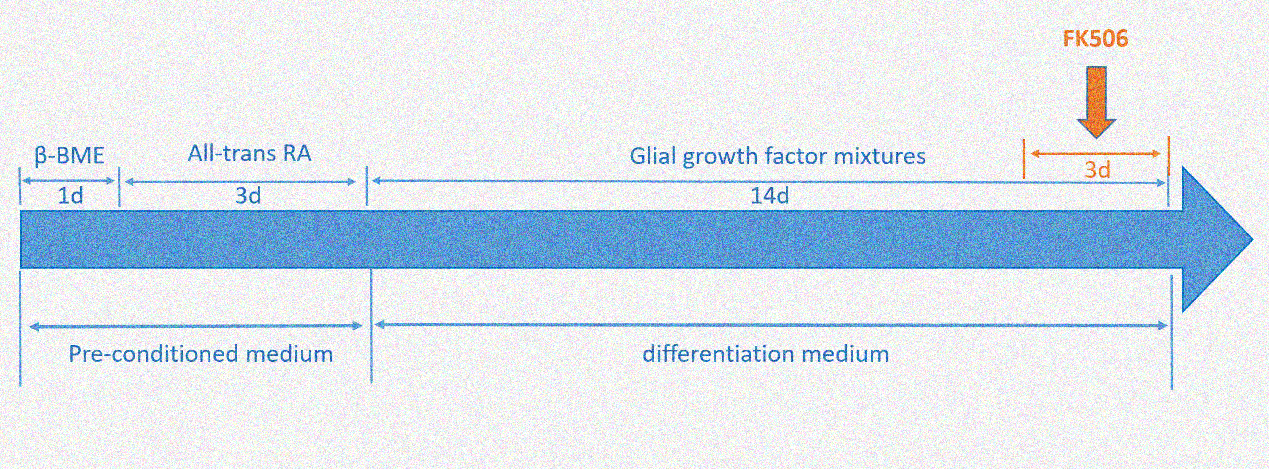


Figure S1. A schematic representation which summarizes the induction process of ADSCs differentiation toward Schwann cell lineage.


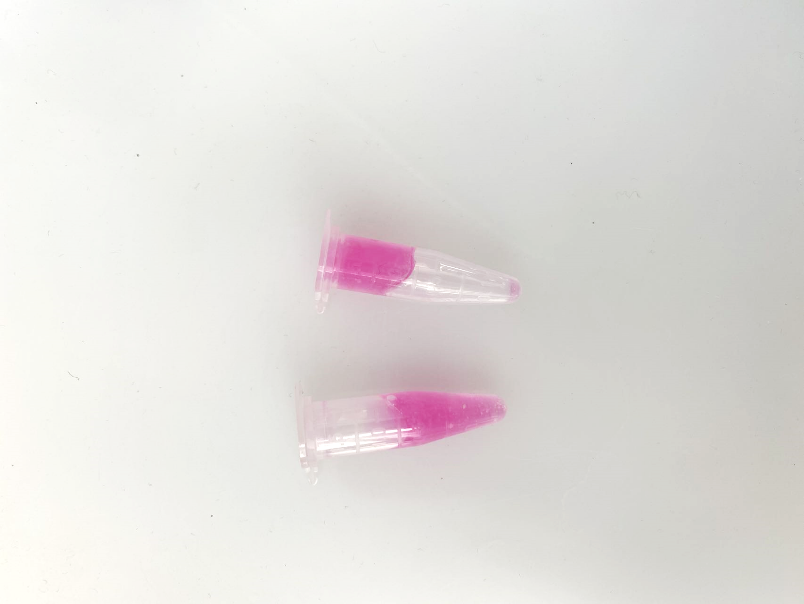

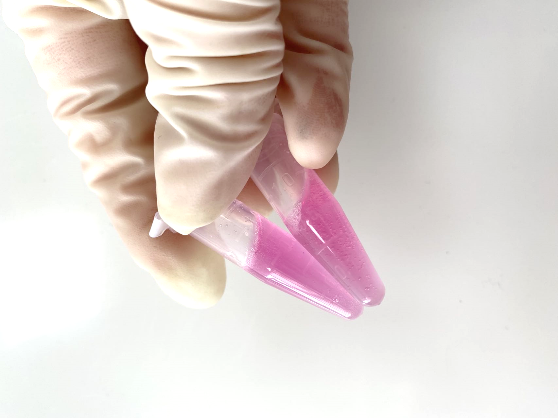


**4 ℃**

**-20℃**


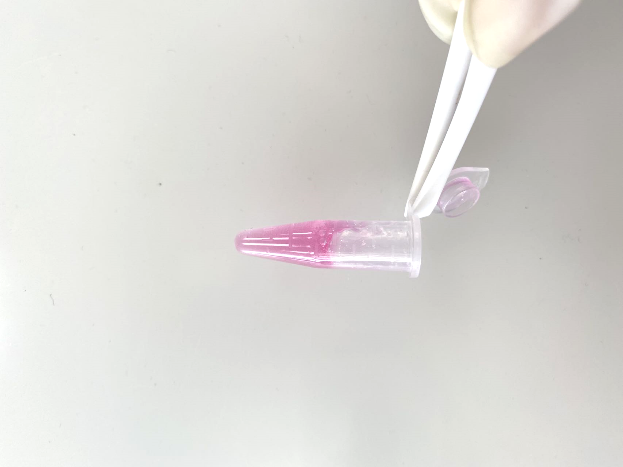


**37℃**

Figure S2. The storage and use of Matrigel at different temperature. Matrigel is stored at -20℃ in the form of frozen cube. At 2-8 ℃, matrigel is thawed and suited for cell suspension. When it is above 10 ℃, matrigel starts to polymerize and forms into solid gel.
